# Supplementary material for: Repeat vaccination reduces antibody affinity maturation across different influenza vaccine platforms in humans
Source: Nat Commun. 2019 Jul 26;10:3338. doi: 10.1038/s41467-019-11296-5 (PMC6659679; doi:10.1038/s41467-019-11296-5)
Supplement: Supplementary file 1 — Supplementary Information [file 41467_2019_11296_MOESM1_ESM.pdf]

Supplementary Information for:

Khurana et. al. Repeat vaccination reduces antibody affinity maturation across different influenza vaccine platforms in humans

Surender Khurana, Ph.D.
Email: Surender.Khurana@fda.hhs.gov

Supplementary Figure 1
Characterization of purified HA proteins for binding to conformation dependent neutralizing MAbs in SPR

Steady-state binding equilibrium analysis of neutralizing MAbs; M19B5 (H1N1 HA1 domain binding antibody that shows HI titer specifically for H1N1pdm09 strain), CR6261 (H1N1 HA stalk domain binding antibody), 3G6 (H3N2 HA1 domain binding antibody that demonstrate HI titer specifically against recent H3N2 strains) and 5A1 (a cross reactive B-strain binding antibody that shows HI titer) to purified HA proteins from H1N1pdm09, H3N2 and B strains immobilized at 1 mcg/ml on a sensor chip through the free amine group in SPR.

Supplementary Figure 1

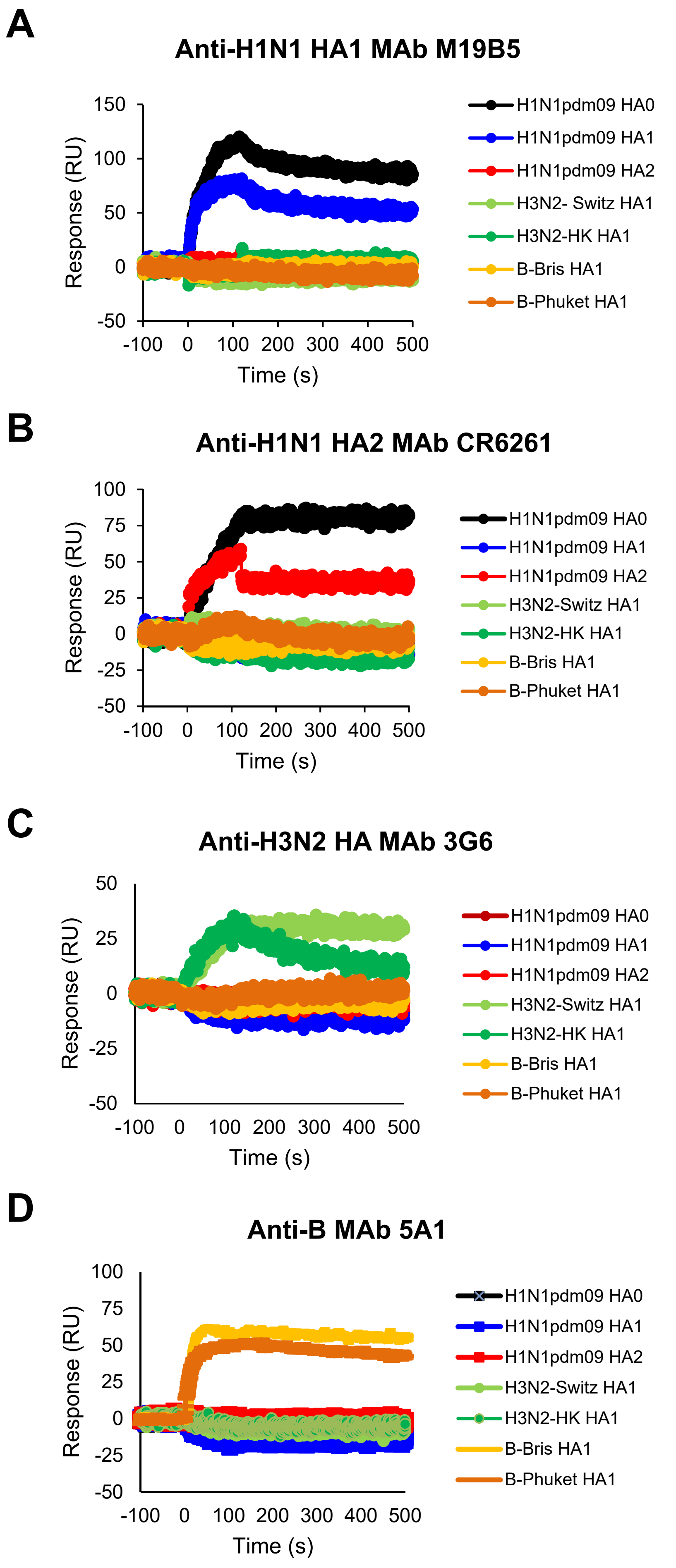

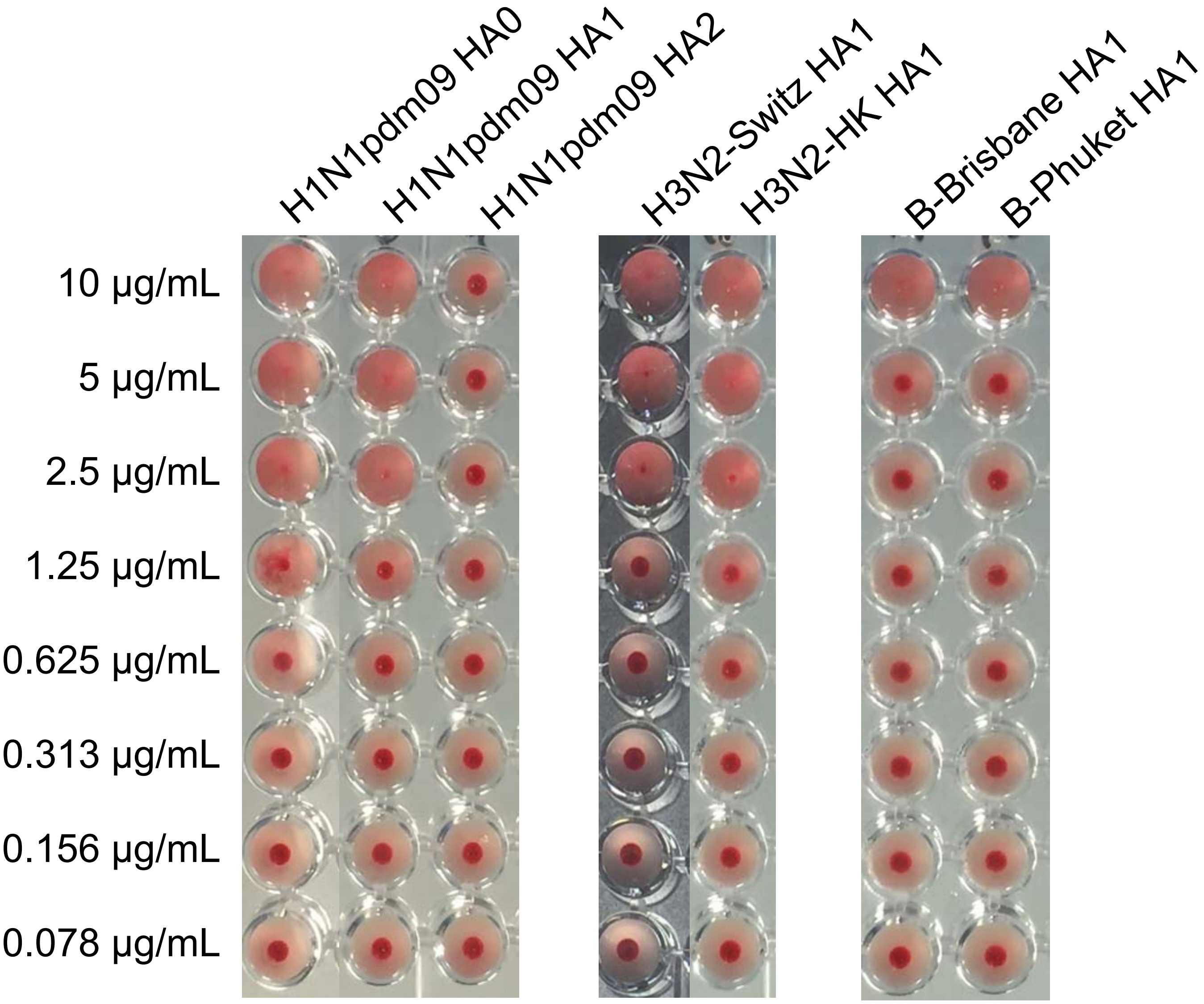

Supplementary Figure 2
Functional characterization of bacterially expressed and purified HA1 and HA2 proteins from *E. coli*
Agglutination of human RBC by HA1 proteins of H1N1pdm09, H3N2 and B strains along with HA0 and HA2 proteins of H1N1pdm09 strain (starting concentration of 10 µg/ml). Serial dilutions of purified HA proteins were mixed with washed RBC and hemagglutination was read after 30 min at RT.

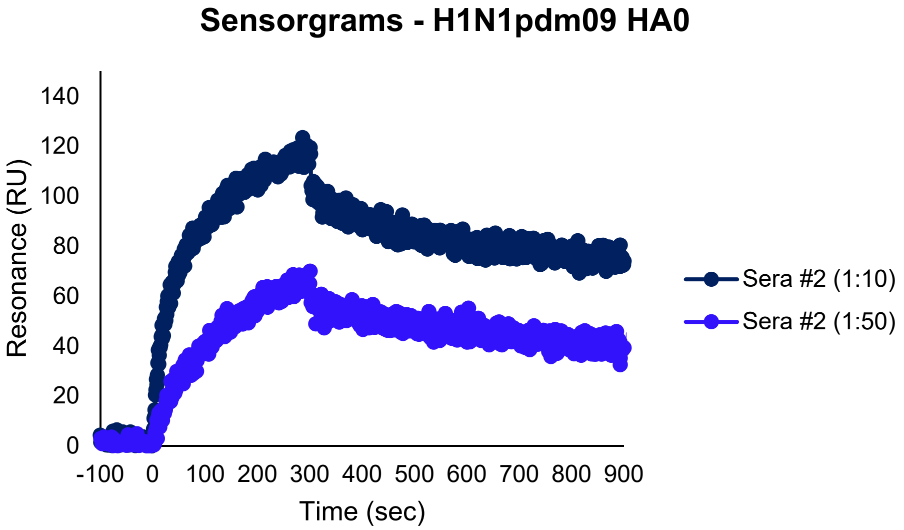

Supplementary Figure 3

Steady-state equilibrium analysis of different dilutions of post-vaccination sera to HA by SPR. Serial dilutions of post-vaccination sera were injected simultaneously onto both H1N1pdm09 HA protein immobilized on a GLC sensor chip and on a surface free of protein (used as a blank). Binding was recorded using BioRad Proteon surface plasmon resonance biosensor instrument. Responses from the protein surface were corrected for the response from the mock surface and for responses from a separate, buffer only injection. Antibody off-rate constants, which describe the fraction of antigen-antibody complexes that decay per second, were determined directly from the serum/plasma sample interaction with rHA using SPR in the dissociation phase only for the sensorgrams with Max RU in the range of 20-150 RU and calculated using the BioRad ProteOn manager software for the heterogeneous sample model

A

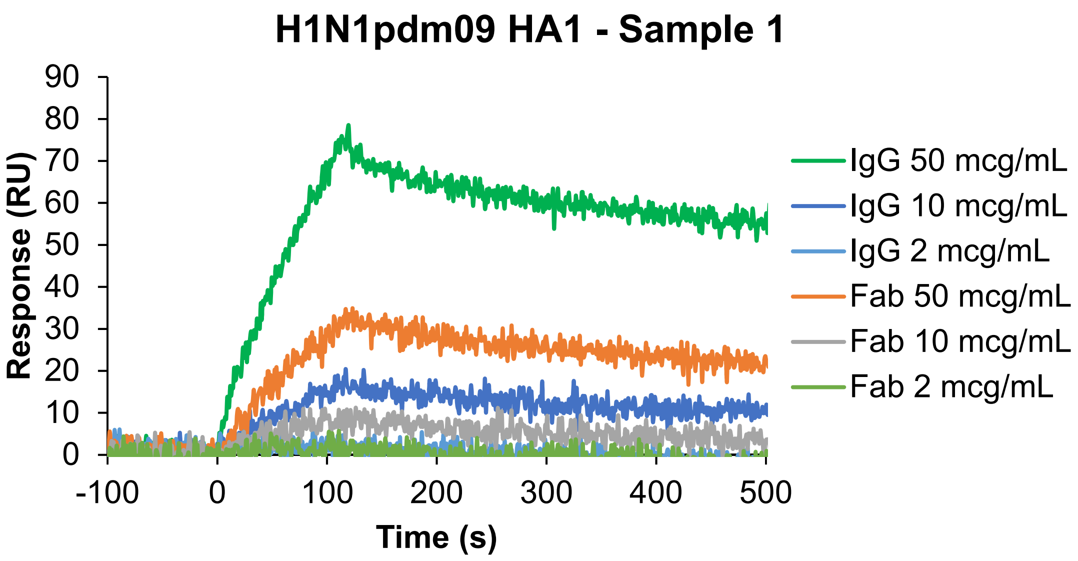

B

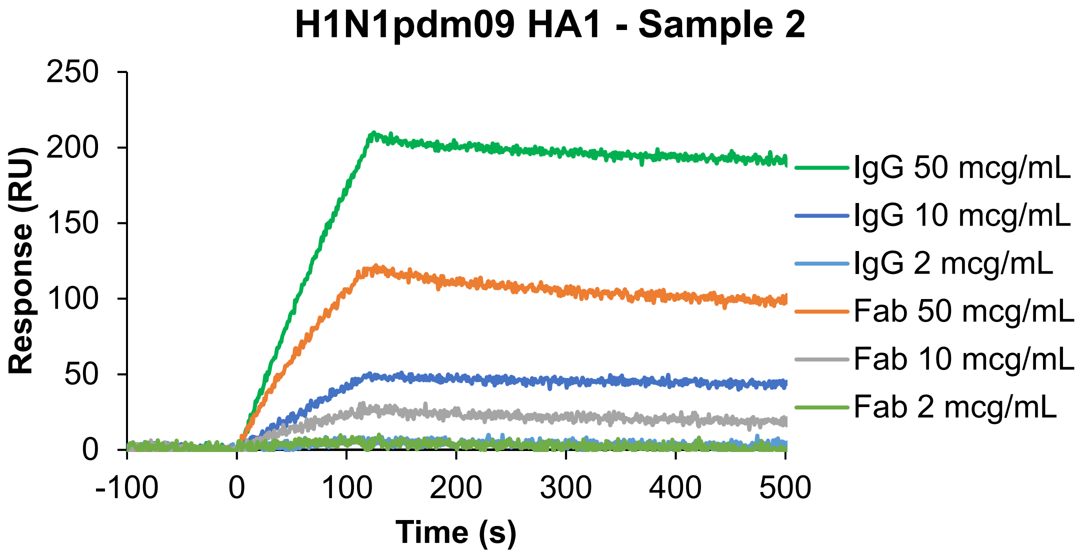

C

	**Off-Rates (1/sec)**			
	**Sample #1**		**Sample #2**	
	50 mcg/ml	10 mcg/ml	50 mcg/ml	10 mcg/ml
**IgG**	2.11E-03	2.02E-03	2.82E-04	2.86E-04
**Fab**	2.13E-03	1.95E-03	4.91E-04	4.92E-04

Supplementary Figure 4

Steady-state equilibrium analysis of purified IgG and Fab to HA by SPR. IgG and Fab were purified from two post-vaccination serum samples (#1 & #2) and serial dilutions of each were injected simultaneously onto H1N1pdm09 HA1 protein immobilized on a sensor chip and on a surface free of protein (used as a blank). Binding was recorded using BioRad ProteOn surface plasmon resonance biosensor instrument. Responses from the protein surface were corrected for the response from the mock surface and for responses from a separate, buffer only injection. Antibody off-rate constants, which describe the fraction of antigen-antibody complexes that decay per second, were determined directly from the IgG and Fab sample interaction with HA1 using SPR in the dissociation phase only for the sensorgrams with Max RU in the range of 20-150 RU and calculated using the BioRad ProteOn manager software for the heterogeneous sample model.

Supplementary Table 1: Demographic and Baseline Characteristics (Excluding 16 repeat subjects)

		Year 1			Year 2		
		FluBlok	FluCelvax	Fluzone	FluBlok	FluCelvax	Fluzone
	N	7	4	4	19	18	17
**Age(yr)**	Mean	23.71	31.50	23.25	28.89	26.89	27.18
	SD	10.29	9.54	7.54	9.49	7.86	9.01
**Sex:**	Female	4 (57%)	2 (50%)	1 (25%)	15 (79%)	13 (72%)	8 (47%)
	Male	3 (43%)	2 (50%)	3 (75%)	4 (21%)	5 (28%)	9 (53%)
**Prior Vacc History**	N	4 (57%)	1 (25%)	2 (50%)	5 (26%)	9 (50%)	2 (12%)
	Y	2 (29%)	1 (25%)	1 (25%)	13 (68%)	9 (50%)	13 (76%)
	unknown	1 (14%)	2 (50%)	1 (25%)	1 (5%)	0 (0%)	2 (12%)
**Baseline HI titer GMT**	H1	97.5	56.6	452.5	133.3	186.6	294.9
	H3	32.8	56.6	47.6	143.4	154.0	204.3
	B	59.4	67.3	67.3	51.6	61.1	76.8

Supplementary Table 2: Day 28 Post-vaccination HI Titers: Comparisons Between Vaccine Groups

		Year 1			Year 2		
**Strain**		**Flublok**	**FluCelvax**	**Fluzone**	**Flublok**	**FluCelvax**	**Fluzone**
**H1**	**GMT[a] (95% CI)**	1721.2 (1027.7, 2882.7)	4537.8 (1517.8, 13566.6)	584.0 (236.4, 1442.4)	1274.3 (784.1, 2070.8)	586.4 (365.1, 941.8)	463.9 (277.6, 775.2)
	**GMT Ratio[b] (95% CI)**	Flublok/FluCelvax	0.38 (0.07, 2.17)		2.17 (0.98, 4.83)		
		Flublok/Fluzone	2.95 (0.61, 14.22)		2.75 (1.11, 6.77)*		
		FluCelvax/Fluzone	7.77 (1.52, 39.83)*		1.26 (0.53, 3.03)		
**H3**	**GMT[a] (95% CI)**	1246.9 (316.1, 4919.0)	516.6 (77.1, 3461.3)	2222.1 (343.5, 14377.3)	662.0 (375.2, 1168.0)	460.3 (260.9, 812.1)	497.9 (283.2, 875.2)
	**GMT Ratio[b] (95% CI)**	Flublok/FluCelvax	2.41 (0.11, 55.20)		1.44 (0.56, 3.69)		
		Flublok/Fluzone	0.56 (0.03, 11.25)		1.33 (0.49, 3.60)		
		FluCelvax/Fluzone	0.23 (0.01, 8.16)		0.92 (0.34, 2.49)		
**B**	**GMT[a] (95% CI)**	257.8 (103.3, 643.2)	407.3 (115.0, 1442.6)	262.3 (75.5, 922.5)	80.1 (52.9, 121.3)	145.9 (96.3, 220.9)	126.2 (83.7, 190.2)
	**GMT Ratio[b] (95% CI)**	Flublok/FluCelvax	0.63 (0.08, 5.03)		0.55 (0.28, 1.09)		
		Flublok/Fluzone	0.98 (0.13, 7.31)		0.64 (0.31, 1.31)		
		FluCelvax/Fluzone	1.55 (0.15, 16.43)		1.16 (0.56, 2.38)		

[a] Geometric mean titers (GMTs) were estimated using an ANOVA model with sex, age, and baseline (day 0) titer as the covariates to adjust for. Analyses were performed on the log transformed titer values, and the estimated least-squares means were back transformed to obtain the GMTs.

[b] Bonferroni adjustment was used to control the type I error for multiple comparisons between vaccine groups.

* Statistically significant fold difference between vaccine groups, i.e., GMT ratio is significantly different from 1.

Supplementary Table 3: Day 28 Post-vaccination HI Titer Fold Change: Comparisons Between Vaccine Groups

		Year 1			Year 2		
**Strain**		**Flublok**	**FluCelvax**	**Fluzone**	**Flublok**	**FluCelvax**	**Fluzone**
**H1**	**GMFC[a] (95% CI)**	19.0†††† (5.0, 72.2)	12.7† (2.0, 80.1)	1.2 (0.2, 7.5)	7.5†††† (4.4, 12.8)	3.0†††† (1.8, 5.2)	1.9† (1.1, 3.2)
	**GMFC Ratio[b] (95% CI)**	Flublok/FluCelvax	1.50 (0.07, 30.20)		**2.48** (1.03, 6.02)*		
		Flublok/Fluzone	15.61 (0.85, 287.46)		**3.93** (1.55, 9.96)***		
		FluCelvax/Fluzone	10.43 (0.34, 319.13)		1.58 (0.63, 4.00)		
**H3**	**GMFC[a] (95% CI)**	31.7†††† (8.2, 122.8)	11.0† (1.7, 71.4)	56.9†††† (9.0, 360.8)	4.0†††† (2.2, 7.4)	2.7††† (1.5, 5.0)	2.8††† (1.5, 5.1)
	**GMFC Ratio[b] (95% CI)**	Flublok/FluCelvax	2.88 (0.14, 60.66)		1.48 (0.54, 4.05)		
		Flublok/Fluzone	0.56 (0.03, 10.70)		1.45 (0.50, 4.19)		
		FluCelvax/Fluzone	0.19 (0.01, 6.17)		0.98 (0.34, 2.83)		
**B**	**GMFC[a] (95% CI)**	3.9† (1.1, 14.1)	7.2† (1.2, 42.1)	4.1 (0.7, 23.1)	1.2 (0.8, 1.9)	2.2†††† (1.4, 3.5)	1.9††† (1.2, 3.0)
	**GMFC Ratio[b] (95% CI)**	Flublok/FluCelvax	0.54 (0.03, 9.60)		0.56 (0.26, 1.18)		
		Flublok/Fluzone	0.97 (0.06, 15.67)		0.64 (0.29, 1.42)		
		FluCelvax/Fluzone	1.78 (0.07, 46.72)		1.15 (0.53, 2.53)		

[a] Geometric mean fold changes (GMFCs) were estimated using an ANOVA model with sex and age as the covariates to adjust for. Analyses were performed on the log transformed fold-rise values, and the estimated least-squares means were back transformed to obtain the GMFCs.

[b] Bonferroni adjustment was used to control the type I error for multiple comparisons between vaccine groups.

* Significant fold difference between vaccine groups, i.e., GMFC ratio is significantly different from 1. * $p<0.05$, ** $p<0.01$, *** $p<0.005$, **** $p<0.001$

† Significant GMFC , i.e., GMFR is significantly different from 1. † $p<0.05$, †† $p<0.01$, ††† $p<0.005$, †††† $p<0.001$

Supplementary Table 4: Day 28 Post-vaccination Max RU: Comparisons Between Vaccine Groups

		Year 1			Year 2		
**Strain**		**Flublok**	**FluCelvax**	**Fluzone**	**Flublok**	**FluCelvax**	**Fluzone**
**H1 HA1**	**GM[a] (95% CI)**	3713.8 (1481.1, 9311.7)	4073.1 (337.0, 49234.7)	1042.9 (180.1, 6040.6)	2410.4 (1728.3, 3361.6)	915.5 (661.2, 1267.5)	508.2 (360.6, 716.2)
	**GM Ratio[b] (95% CI)**	Flublok/FluCelvax	0.91 (0.02, 36.29)		**2.63** (1.52, 4.57)****		
		Flublok/Fluzone	3.56 (0.21, 61.25)		**4.74** (2.59, 8.70)****		
		FluCelvax/Fluzone	3.91 (0.06, 250.61)		1.80 (1.00, 3.25)		
**H1 HA2**	**GM[a] (95% CI)**	2478.1 (2003.2, 3065.7)	3483.6 (2106.9, 5759.9)	2767.2 (1923.0, 3982.0)	2099.5 (1761.8, 2501.8)	1889.2 (1588.6, 2246.7)	1684.5 (1406.6, 2017.2)
	**GM Ratio[b] (95% CI)**	Flublok/FluCelvax	0.71 (0.33, 1.55)		1.11 (0.83, 1.50)		
		Flublok/Fluzone	0.90 (0.50, 1.62)		1.25 (0.90, 1.72)		
		FluCelvax/Fluzone	1.26 (0.62, 2.57)		1.12 (0.82, 1.53)		
**H1 HA0**	**GM[a] (95% CI)**	1459.9 (678.3, 3142.2)	1037.8 (200.5, 5373.0)	632.1 (180.1, 2218.8)	1802.6 (1343.3, 2419.0)	1155.7 (868.2, 1538.3)	678.7 (499.1, 923.0)
	**GM Ratio[b] (95% CI)**	Flublok/FluCelvax	1.41 (0.11, 18.80)		1.56 (0.96, 2.53)		
		Flublok/Fluzone	2.31 (0.26, 20.21)		**2.66** (1.54, 4.58)****		
		FluCelvax/Fluzone	1.64 (0.15, 17.91)		1.70 (1.01, 2.88)*		
**H3 HA1**	**GM[a] (95% CI)**	4650.2 (1581.4, 13674.3)	4398.1 (336.5, 57486.9)	7518.5 (1329.6, 42513.3)	2662.4 (1772.8, 3998.5)	1616.1 (1083.2, 2411.1)	1760.6 (1153.0, 2688.3)
	**GM Ratio[b] (95% CI)**	Flublok/FluCelvax	1.06 (0.02, 66.87)		1.65 (0.84, 3.22)		
		Flublok/Fluzone	0.62 (0.04, 10.91)		1.51 (0.72, 3.17)		
		FluCelvax/Fluzone	0.58 (0.02, 20.66)		0.92 (0.44, 1.91)		
**B HA1**	**GM[a] (95% CI)**	1372.1 (417.2, 4512.1)	9831.9 (475.6, 203271.9)	2714.5 (395.7,18620.5)	1681.8 (1191.4, 2374.1)	2750.2 (1958.7, 3861.4)	2070.0 (1443.1, 2969.2)
	**GM Ratio[b] (95% CI)**	Flublok/FluCelvax	0.14 (0.00, 11.09)		0.61 (0.35, 1.08)		
		Flublok/Fluzone	0.51 (0.02, 11.29)		0.81 (0.43, 1.52)		
		FluCelvax/Fluzone	3.62 (0.06, 217.41)		1.33 (0.71, 2.47)		

[a] Geometric means (GMs) were estimated using an ANOVA model with sex, age, and baseline (day 0) value as the covariates to adjust for. Analyses were performed on the log transformed values, and the estimated least-squares means were back transformed to obtain the GMs.

[b] Bonferroni adjustment was used to control the type I error for multiple comparisons between vaccine groups.

* Statistically significant fold difference between vaccine groups, i.e., GM ratio is significantly different from 1.

Supplementary Table 5: Day 28 Post-vaccination Max RU Fold Change: Comparisons Between Vaccine Groups

		Year 1			Year 2		
Strain		Flublok	FluCelvax	Fluzone	Flublok	FluCelvax	Fluzone
H1 HA1	GMFC[a] (95% CI)	**11.7**†††† (4.8, 28.6)	**6.3**†† (1.8, 21.7)	1.2 (0.3, 3.9)	**8.5**†††† (6.0, 12.1)	**2.9**†††† (2.0, 4.1)	**1.7**††† (1.2, 2.4)
	GMFC Ratio[b] (95% CI)	Flublok/FluCelvax	1.85 (0.25, 13.79)		**2.97** (1.65, 5.36)****		
		Flublok/Fluzone	10.07 (1.44, 70.55)*		**4.97** (2.67, 9.23)****		
		FluCelvax/Fluzone	5.44 (0.55, 52.98)		1.67 (0.90, 3.10)		
H1 HA2	GMFC[a] (95% CI)	1.2 (1.0, 1.4)	1.3† (1.0, 1.6)	1.3† (1.1, 1.7)	**1.5**†††† (1.2, 1.8)	1.2† (1.0, 1.4)	1.0 (0.9, 1.3)
	GMFC Ratio[b] (95% CI)	Flublok/FluCelvax	0.90 (0.62, 1.32)		1.22 (0.90, 1.65)		
		Flublok/Fluzone	0.86 (0.60, 1.25)		1.41 (1.03, 1.93)*		
		FluCelvax/Fluzone	0.96 (0.62, 1.48)		1.15 (0.84, 1.58)		
H1 HA0	GMFC[a] (95% CI)	**4.3**†††† (2.4, 7.6)	**3.7**††† (1.7, 8.3)	1.8 (0.8, 3.9)	**6.0**†††† (4.2, 8.6)	**3.2**†††† (2.3, 4.7)	**1.8**††† (1.2, 2.5)
	GMFC Ratio[b] (95% CI)	Flublok/FluCelvax	1.15 (0.31, 4.23)		1.84 (1.01, 3.37)*		
		Flublok/Fluzone	2.41 (0.68, 8.53)		**3.40** (1.80, 6.39)****		
		FluCelvax/Fluzone	2.10 (0.47, 9.28)		1.84 (0.98, 3.46)		
H3 HA1	GMFC[a] (95% CI)	**5.8**†††† (2.5, 13.6)	2.4 (0.8, 7.9)	**11.7**†††† (3.7, 37.2)	**3.8**†††† (2.4, 6.2)	**2.3**††† (1.4, 3.7)	**2.1**††† (1.3, 3.5)
	GMFC Ratio[b] (95% CI)	Flublok/FluCelvax	2.37 (0.35, 16.09)		1.68 (0.76, 3.74)		
		Flublok/Fluzone	0.50 (0.08, 3.17)		1.78 (0.77, 4.12)		
		FluCelvax/Fluzone	0.21 (0.02, 1.85)		1.06 (0.46, 2.44)		
B HA1	GMFC[a] (95% CI)	2.3 (0.9, 5.8)	3.4 (0.9, 12.5)	3.0 (0.8, 10.8)	1.8† (1.2, 2.9)	**3.0**†††† (1.9, 4.7)	**1.9**†† (1.2, 3.0)
	GMFC Ratio[b] (95% CI)	Flublok/FluCelvax	0.67 (0.08, 5.57)		0.62 (0.29, 1.35)		
		Flublok/Fluzone	0.76 (0.10, 5.94)		0.97 (0.43, 2.20)		
		FluCelvax/Fluzone	1.42 (0.10, 20.00)		1.56 (0.69, 3.52)		

[a] Geometric mean fold changes (GMFCs) were estimated using an ANOVA model with sex and age as the covariates to adjust for. Analyses were performed on the log transformed fold-rise values, and the estimated least-squares means were back transformed to obtain the GMFCs.

[b] Bonferroni adjustment was used to control the type I error for multiple comparisons between vaccine groups.

* Significant fold difference between vaccine groups, i.e., GMFC ratio is significantly different from 1. * p<0.05, ** p<0.01, *** p<0.005, **** p<0.001

† Significant GMFC , i.e., GMFR is significantly different from 1. † p<0.05, †† p<0.01, ††† p<0.005, †††† p<0.001

Supplementary Table 6: Day 28 Post-vaccination Kd (per Sec): Comparisons Between Vaccine Groups

		Year 1			Year 2		
**Strain**		**Flublok**	**FluCelvax**	**Fluzone**	**Flublok**	**FluCelvax**	**Fluzone**
**H1 HA1**	**GM[a] (95% CI)**	0.00052 (0.00025, 0.00106)	0.00090 (0.00033, 0.00247)	0.00034 (0.00010, 0.00118)	0.00059 (0.00036, 0.00098)	0.00096 (0.00058, 0.00157)	0.00128 (0.00077, 0.00212)
	**GM Ratio[b] (95% CI)**	Flublok/FluCelvax	0.57 (0.12, 2.70)		0.62 (0.27, 1.42)		
		Flublok/Fluzone	1.51 (0.20, 11.34)		0.46 (0.19, 1.14)		
		FluCelvax/Fluzone	2.63 (0.25, 27.14)		0.75 (0.31, 1.79)		
**H1 HA2**	**GM[a] (95% CI)**	0.00026 (0.00013, 0.00053)	0.00029 (0.00011, 0.00078)	0.00030 (0.00012, 0.00075)	0.00025 (0.00017, 0.00037)	0.00031 (0.00020, 0.00046)	0.00050 (0.00033, 0.00076)
	**GM Ratio[b] (95% CI)**	Flublok/FluCelvax	0.92 (0.17, 5.02)		0.82 (0.42, 1.60)		
		Flublok/Fluzone	0.87 (0.19, 3.94)		0.50 (0.25, 1.01)		
		FluCelvax/Fluzone	0.95 (0.16, 5.54)		0.61 (0.28, 1.30)		
**H1 HA0**	**GM[a] (95% CI)**	0.00035 (0.00023, 0.00053)	0.00018 (0.00010, 0.00031)	0.00054 (0.00030, 0.00095)	0.00024 (0.00018, 0.00031)	0.00021 (0.00016, 0.00028)	0.00033 (0.00025, 0.00043)
	**GM Ratio[b] (95% CI)**	Flublok/FluCelvax	2.01 (0.81, 4.98)		1.11 (0.71, 1.74)		
		Flublok/Fluzone	0.66 (0.26, 1.68)		0.73 (0.45, 1.16)		
		FluCelvax/Fluzone	0.33 (0.11, 0.95)*		0.65 (0.41, 1.04)		
**H3 HA1**	**GM[a] (95% CI)**	0.00030 (0.00012, 0.00075)	0.00088 (0.00025, 0.00312)	0.00064 (0.00018, 0.00223)	0.00082 (0.00048, 0.00140)	0.00092 (0.00054, 0.00157)	0.00144 (0.00084, 0.00247)
	**GM Ratio[b] (95% CI)**	Flublok/FluCelvax	0.34 (0.04, 2.69)		0.89 (0.37, 2.16)		
		Flublok/Fluzone	0.47 (0.06, 3.50)		0.57 (0.22, 1.48)		
		FluCelvax/Fluzone	1.38 (0.13, 14.43)		0.64 (0.25, 1.66)		
**B HA1**	**GM[a] (95% CI)**	0.00076 (0.00019, 0.00300)	0.00052 (0.00008, 0.00353)	0.00173 (0.00026, 0.01135)	0.00326 (0.00186, 0.00570)	0.00126 (0.00072, 0.00220)	0.00304 (0.00176, 0.00527)
	**GM Ratio[b] (95% CI)**	Flublok/FluCelvax	1.46 (0.06, 33.33)		2.59 (1.03, 6.53)*		
		Flublok/Fluzone	0.44 (0.02, 9.05)		1.07 (0.41, 2.83)		
		FluCelvax/Fluzone	0.30 (0.01, 10.94)		0.41 (0.16, 1.09)		

[a] Geometric means (GMs) were estimated using an ANOVA model with sex, age, and baseline (day 0) value as the covariates to adjust for. Analyses were performed on the log transformed values, and the estimated least-squares means were back transformed to obtain the GMs.

[b] Bonferroni adjustment was used to control the type I error for multiple comparisons between vaccine groups.

* Statistically significant fold difference between vaccine groups, i.e., GM ratio is significantly different from 1.

Supplementary Table 7: Day 28 Post-vaccination Kd (per Sec) Fold Change: Comparisons Between Vaccine Groups

		Year 1			Year 2		
**Strain**		**Flublok**	**FluCelvax**	**Fluzone**	**Flublok**	**FluCelvax**	**Fluzone**
**H1 HA1**	**GMFC[a] (95% CI)**	**0.08**†††† (0.03, 0.23)	**0.10**†† (0.02, 0.46)	0.35 (0.08, 1.53)	**0.14**†††† (0.08, 0.24)	**0.28**†††† (0.16, 0.50)	0.48† (0.27, 0.84)
	**GMFC Ratio[b] (95% CI)**	Flublok/FluCelvax	0.75 (0.07, 8.59)		0.48 (0.19, 1.25)		
		Flublok/Fluzone	0.22 (0.02, 2.33)		0.29 (0.11, 0.78)**		
		FluCelvax/Fluzone	0.29 (0.02, 4.72)		0.60 (0.22, 1.61)		
**H1 HA2**	**GMFC[a] (95% CI)**	0.60 (0.29, 1.26)	0.40 (0.14, 1.09)	0.54 (0.20, 1.47)	**0.44**†††† (0.28, 0.69)	0.69 (0.44, 1.08)	0.67 (0.43, 1.04)
	**GMFC Ratio[b] (95% CI)**	Flublok/FluCelvax	1.53 (0.29, 7.92)		0.63 (0.30, 1.33)		
		Flublok/Fluzone	1.12 (0.23, 5.50)		0.66 (0.30, 1.43)		
		FluCelvax/Fluzone	0.73 (0.11, 4.76)		1.03 (0.47, 2.26)		
**H1 HA0**	**GMFC[a] (95% CI)**	**0.26**††† (0.13, 0.52)	**0.14**†††† (0.05, 0.36)	0.66 (0.26, 1.71)	**0.40**†††† (0.24, 0.66)	**0.50**†† (0.30, 0.82)	0.75 (0.46, 1.22)
	**GMFC Ratio[b] (95% CI)**	Flublok/FluCelvax	1.86 (0.39, 8.93)		0.81 (0.36, 1.87)		
		Flublok/Fluzone	0.39 (0.09, 1.78)		0.54 (0.23, 1.29)		
		FluCelvax/Fluzone	0.21 (0.04, 1.25)		0.66 (0.28, 1.58)		
**H3 HA1**	**GMFC[a] (95% CI)**	**0.04**†††† (0.01, 0.11)	**0.10**†† (0.03, 0.44)	**0.07**††† (0.02, 0.30)	**0.30**†††† (0.17, 0.54)	**0.34**†††† (0.19, 0.60)	0.67 (0.38, 1.18)
	**GMFC Ratio[b] (95% CI)**	Flublok/FluCelvax	0.39 (0.04, 3.99)		0.91 (0.35, 2.36)		
		Flublok/Fluzone	0.55 (0.06, 5.27)		0.46 (0.17, 1.25)		
		FluCelvax/Fluzone	1.42 (0.10, 20.00)		0.50 (0.18, 1.37)		
**B HA1**	**GMFC[a] (95% CI)**	0.16† (0.04, 0.66)	0.09† (0.01, 0.65)	0.42 (0.06, 2.84)	0.57 (0.30, 1.08)	**0.22**†††† (0.12, 0.42)	0.51† (0.27, 0.95)
	**GMFC Ratio[b] (95% CI)**	Flublok/FluCelvax	1.73 (0.07, 41.28)		2.56 (0.89, 7.36)		
		Flublok/Fluzone	0.39 (0.02, 8.37)		1.13 (0.37, 3.43)		
		FluCelvax/Fluzone	0.22 (0.01, 8.25)		0.44 (0.15, 1.33)		

[a] Geometric mean fold changes (GMFCs) were estimated using an ANOVA model with sex and age as the covariates to adjust for. Analyses were performed on the log transformed fold-rise values, and the estimated least-squares means were back transformed to obtain the GMFCs.

[b] Bonferroni adjustment was used to control the type I error for multiple comparisons between vaccine groups.

* Significant fold difference between vaccine groups, i.e., GMFC ratio is significantly different from 1. * p<0.05, ** p<0.01, *** p<0.005, **** p<0.001

† Significant GMFC , i.e., GMFR is significantly different from 1. † p<0.05, †† p<0.01, ††† p<0.005, †††† p<0.001

Supplementary Table 8: Effect of prior vaccination on HI fold change for each vaccine group: inactivated vs none, Yr 1 and Yr 2 combined (for Figure 5, A,B,C)

Strain	Vaccine	Prior Vaccination		Ratio (Inactivated/none)	p-value
		None	Inactivated		
H1N1	FluBlok	34.6	5.7	0.16	0.0002****
	FluCelvax	7.0	2.6	0.38	0.0492*
	Fluzone	2.0	1.6	0.82	0.7473
H3N2	FluBlok	20.2	4.0	0.20	0.0047***
	FluCelvax	5.7	2.0	0.35	0.0759
	Fluzone	11.3	2.8	0.25	0.0623
B	FluBlok	4.0	1.2	0.29	0.0033***
	FluCelvax	7.0	1.2	0.18	0.0001****
	Fluzone	3.4	1.6	0.49	0.1846

Supplementary Table 9: Effect of prior vaccination on off-rate fold change for each vaccine group: inactivated vs none, Yr 1 and Y2 combined (for Figure 5, D-K)

Strain	Vaccine	Prior Vaccination		Ratio (Inactivated/none)	*p-value*[b] (inactivated/ none)
		None FC (*p-value*[a])	Inactivated FC (*p-value*)		
H1 HA1	FluBlok	0.04†††† *<.0001*	0.22†††† *<.0001*	5.94****	*<.0001*
	FluCelvax	0.10†††† *<.0001*	0.57† *0.0411*	5.76****	*<.0001*
	Fluzone	0.10†††† *<.0001*	0.59† *0.0252*	6.05****	*0.0005*
H1 HA2	FluBlok	0.52† *0.0289*	0.59† *0.0314*	1.15	*0.7132*
	FluCelvax	0.66 *0.1424*	0.78 *0.3853*	1.19	*0.6664*
	Fluzone	0.53 *0.1588*	0.72 *0.1737*	1.36	*0.5443*
H1 HA0	FluBlok	0.15†††† *<.0001*	0.54† *0.0165*	3.62***	*0.0019*
	FluCelvax	0.25†††† *<.0001*	0.69 *0.2095*	2.71*	*0.0193*
	Fluzone	0.79 *0.6030*	0.72 *0.1901*	0.92	*0.8702*
H3 HA1	FluBlok	0.03††† *<.0001*	0.43†††† *0.0009*	12.82****	*<.0001*
	FluCelvax	0.14†††† *<.0001*	0.70 *0.2094*	5.05****	*0.0002*
	Fluzone	0.23††† *0.0017*	0.62† *0.0497*	2.72	*0.0542*
B HA1	FluBlok	0.10†††† *<.0001*	0.74 *0.3325*	7.55****	*0.0002*
	FluCelvax	0.07†††† *<.0001*	0.56 *0.1265*	8.55****	*0.0001*
	Fluzone	0.23† *0.0150*	0.66 *0.1847*	2.87	*0.1195*

[a] p-value for Ho: fold change = 1 (ie, no change) † p<0.05, †† p<0.01, ††† p<0.005, †††† p<0.001

[b] p-value for Ho: ratio of fold change (inactivated/none) =1 (ie, no effect of prior vaccination) * p<0.05, **p<0.01, ***p<0.005, ****p<0.001
